# Supplementary material for: The effect of psychological distress on IVF outcomes: Reality or speculations?
Source: PLoS One. 2020 Dec 14;15(12):e0242024. doi: 10.1371/journal.pone.0242024 (PMC7735622; doi:10.1371/journal.pone.0242024)
Supplement: S1 Table — (DOCX) [file pone.0242024.s001.docx]

**S1 Table.** Intercorrelations between Fertility Problem Inventory (FPI) Subscales.

| FPI scales | Social concern | Sexual concern | Relationship concern | Need for parenthood | Rejection of childfree lifestyle | Global stress |
| --- | --- | --- | --- | --- | --- | --- |
| Social concern | 1 |  |  |  |  |  |
| Sexual concern | 0.60*** | 1 |  |  |  |  |
| Relationship concern | 0.59*** | 0.70*** | 1 |  |  |  |
| Need for parenthood | -0.18** | 0.05 | -0.13* | 1 |  |  |
| Rejection of childfree lifestyle | 0.39*** | 0.35*** | 0.38*** | 0.03 | 1 |  |
| Global stress | 0.69*** | 0.80*** | 0.77*** | 0.27*** | 0.70*** | 1 |

*p<0.05, **p<0.01, ***p<0.001
