# Supplementary material for: The effect of psychological distress on IVF outcomes: Reality or speculations?
Source: PLoS One. 2020 Dec 14;15(12):e0242024. doi: 10.1371/journal.pone.0242024 (PMC7735622; doi:10.1371/journal.pone.0242024)
Supplement: S2 Table — (DOCX) [file pone.0242024.s002.docx]

**S2 Table.** Correlations of Fertility Problem Inventory (FPI) Scale with Depression (CES-D Scale), State Anxiety (STAI-S) and Trait Anxiety (STAI-T) Scales.

|  | Social concern | Sexual concern | Relationship concern | Need for parenthood | Rejection of childfree lifestyle | Global stress |
| --- | --- | --- | --- | --- | --- | --- |
| CES-D | 0.08 | 0.10 | 0.12* | -0.08 | 0.12 | 0.10 |
| STAI-S | 0.04 | 0.20** | 0.06 | 0.14* | -0.05 | 0.11 |
| STAI-T | -0.07 | 0.08 | -0.02 | 0.20** | -0.04 | 0.05 |

*p<0.05, **p<0.01, ***p<0.
