# Supplementary material for: The effect of psychological distress on IVF outcomes: Reality or speculations?
Source: PLoS One. 2020 Dec 14;15(12):e0242024. doi: 10.1371/journal.pone.0242024 (PMC7735622; doi:10.1371/journal.pone.0242024)
Supplement: Attachment — Submitted filename: Review comments.odt [file pone.0242024.s003.odt]

Review comments


Title: The effect of psychological distress on IVF outcomes: reality or speculations?

This study investigates the rates of depression, anxiety and stress in women undergoing fertility treatement in Kazakhstan. Furthermore the present study explored if increased levels of depressive symptoms and anxiety influenced pregnancy outcome. Previously there haven't been exact numbers of how many women are burdened by the infertility or treatment nor if it has had any negative influence on pregnancy rates for women in the region of Kazakhstan, so it is very admirable that the authors seek to investigate. However there are some structural and language issues that need attention. 

General comments: There are places in the manuscript where the argumentation is very sparse. It would be better if you leave out some of the points and build the understanding for the points left rather than a lot of points with no reasoning. Then it becomes difficult to see the relevance of the point. It could be interesting if the authors disclosed a little more of the importance of why infertility might be so devastating for women in the region of central asia - some information is discussed in the end of the manuscript. I find this information very important and interesting. Also, discussing admissability to treatment.


Introduction

L. 72-74: It would be good if the authors explained from where they got these definitions of infertility, although they put in a reference, as there are differenct definitions according to country and region.

L. 79-80: I would advise the authors to discuss the terms stress and distress, and when to use  the nomination. It seems that due to sadness and dissapointment one would experience distress (emotional burden), whereas treatment in itself can be stressing and probably lead to distress.

L. 81-84: The aregumenation is a little too superficial. I agree that stress and distress may influence autominic and endocrine responses, and this, is defintely very interesting. However, the argumentation lacks examples or referral to the specific studies - the 'how'.

L. 85-88: This is true - but this paragraph seem to be associated with former paragraphs explaining that couples are distressed or that infertility and fertility treatment might be stressing. Be aware that you don't jump around with different themes. 

L.103: What does REI stand for?

Methods

L- 112: How many patients were approached? Did the women themselves decide if they wanted to take part in teh study?

L. 118: You might want to specify that two clinics were in the same city??? 3 clinics - two cities.

L. 125: When did they fill-out the baseine questionnaire?

L. 129: What kind of medical history information was gathered? Previous depressions, anxiety - treatments? Prescribed medication?

L.140-146: Why did you choose CES-D? And what is the average score of the Kazakhstan population? or regional data?

L.187: I believe that The Results section should be a header in itself and not presented under 'methods'.

Results

L. 189: How many did you approach - how many declined to particioate? Do you know why? A flowchart will give us a nice overview.

L. 190: definition of normal BMI (also in a cultural perspective). Also it is unclear, if women who conceived were in first, second or more cycles. It would be beneficial to add this information to your table (the frequency of pregnacy rate for each cycle).

L. 220-222: from what perspective? Do you have any regional data to compare with? Again a table with an overview of the descriptive reginal data would make it much easier to understand  the differences. 

L.230-233: I am a little usure that I understand this paragraph correctly. If you controlled for the variables mentionned then the association between FPI global stress was still significant or not significant for pregnancy outcome? You may want to consider 'main' analyses and then explorative analyses e.g. looking into specific domains association with pregnancy. It is a little muddled. 

Discussion

Overall, the discussion is long. You may want to shorten it a bit. 

L.251-261: This is a very interesting discussion point indeed, however as you don't yourselves present any biological/physiological data it seems a little odd to present it here. You are speculating whether this might be an explanation for your findings.

L.272-281: What is the main reason for this paragraph? You might want to leave it out. As it doesn't add to your main objective.

L. 301: If you did power calculations accordingly to the number of participants you needed, it would be a good idea to present this information in the Methods section, as this is defintely a strength.

L. 313-315: This is an interesting result, wy isn't this dicussed further?
